# Anesthesia management experience for pediatric day-case PDA ligation under thoracoscopy assisted by a robot: a retrospective study

**DOI:** 10.1186/s13019-023-02471-3

**Published:** 2023-12-19

**Authors:** Huixia Cao, Jinpeng Qiu, Yaoqin Hu, Wenfang Huang, Xiwang Liu, Haiyan Jin

**Affiliations:** 1 Department of Anesthesiology, Children’s Hospital, Zhejiang University School of Medicine, National Clinical Research Center for Child Health, 3333 Binsheng Road, Binjiang District, Hangzhou, 310052 China; 2 Department of Cardiac Surgery, Children’s Hospital, Zhejiang University School of Medicine, National Clinical Research Center for Child Health, Hangzhou, 310052 China

**Keywords:** Robot-assisted thoracoscopy, Day-case, Anesthesia, Patent ductus arteriosus

## Abstract

**Background:**

To summarize the anesthesia management experience for pediatric day-case patent ductus arteriosus (PDA) ligation under robot-assisted thoracoscopy and explore the key points of anesthesia management for this procedure.

**Methods:**

The clinical data of 72 pediatric patients who underwent robot-assisted thoracoscopic day-case PDA ligation at the Children’s Hospital, Zhejiang University School of Medicine from April 2021 to February 2023 were retrospectively analyzed. 0.3% ropivacaine local infiltration combined with S-ketamine 0.2 mg/kg intravenous injection was used for postoperative analgesia The patient’s basic information and intraoperative conditions were analyzed, which included gender, age, weight, surgery time, anesthesia time, extubation time, intraoperative blood loss, MAP before pneumothorax, PaCO2 before pneumothorax, etc. Postoperative conditions were also monitored, such as PACU stay time, agitation during the recovery period, pain, and the incidence of nausea and vomiting. After discharge, the recovery status was assessed.

**Results:**

A total of 70 pediatric patients who met the criteria for day-case PDA ligation were included in this study. Before the occurrence of pneumothorax, the mean arterial pressure (MAP) of these 70 patients was 69.58 ± 12.52 mmHg, and during controlled hypotension, the MAP was 54.96 ± 11.23 mmHg. Before the occurrence of pneumothorax, the partial pressure of carbon dioxide (PaCO_2_) was 38.69 ± 3.38 mmHg, and during controlled hypotension, the PaCO_2_ was 51.42 ± 4.05 mmHg. Three cases experienced agitation during the recovery period, and four cases had mild pain, but there was no moderate or severe pain, nausea, or vomiting. Only 1 case of postoperative respiratory tract infection and 1 case of postoperative pneumothorax occurred. Within 30 days after discharge, the unplanned revisit rate, unplanned readmission rate, and surgical wound infection rate were all 0. The residual shunt rate detected by echocardiography was 0 after 1 month.

**Conclusions:**

The children under the robot-assisted thoracoscopic day case PDA surgeries in this study have limited trauma, little bleeding, and little postoperative pain, though still at a risk of respiratory infection and pneumothorax.

## Background

Day-case surgery can shorten the hospital stay of children, reduce medical costs, ease the financial burden on the families and health insurance of children [1]. The concept of day-case surgery is well established in developed countries but is still in its infancy in developing countries such as China. A survey conducted in 19 countries in 2006 found an extremely wide variation in the percentage of day cases, with 10% in Poland and more than 80% in the United States and Canada [2]. In 2020, the number of day-case surgery in tertiary public hospitals on China will increase by 6.44% compared to 2019, and the proportion of day-case surgery to elective surgery will be 10.85%, an increase of 1.93% points compared to 2019 [3].

In conventional surgery, generally one or more days should be spent in advance of surgery to complete the initial evaluation of surgery, and one or more days after hospitalization for recovery. In the day-case surgery mode, the initial diagnosis, examination and evaluation, health education, are completed before admission, the children can be hospitalized to discharge within 24 h under the premise of ensuring the safety of patients. The duration of surgery was the most important criterion for patient selection for day-case surgery, followed by clinical status and comorbidities; and surgery lasting within 2 h could be considered for day-case surgery [4, 5].

Although day-case surgery is applicable to a broad range of procedures, there has been limited research on performing day-case closure of patent ductus arteriosus (PDA) in children, and experience with anesthesia management is still immature in this context [6]. Studies done on video-assisted thoracoscopic surgery-PDA ligation showed better outcomes when compared to open thoracotomies [7]. Ying et al. reported that all the 106 cases underwent robotically assisted surgery for PDA ligation, and good efficiency were achieved with a short operation time, little bleeding, and a short postoperative hospital stays were 1–3 days [8]. Robotic surgery for PDA ligation in children can be considered for its safety, effeciency, and dependable surgical method [8]. However, the day-case surgery for pediatric PDA ligation using robot-assisted thoracoscopy were rarely reported.

The purpose of this study is to present a summary of the experience of day-case surgery for pediatric PDA ligation using robot-assisted thoracoscopy.

## Data and methods

The medical records of 70 children who received robot-assisted thoracoscopic PDA closure surgery in the day-case surgery unit at the Children’s Hospital, Zhejiang University School of Medicine, were retrospectively analyzed by this study.

### Clinical data

The clinical data of 72 pediatric patients who underwent robot-assisted thoracoscopic closure of PDA during 24-hour day surgery from April 2021 to February 2023 were retrospectively collected after approval by the Medical Ethics Committee of the Children’s Hospital, Zhejiang University School of Medicine (ethics number: 2023-IRB-0053-P-01). Written informed consent was waved by Medical Ethics Committee because this was a retrospective study. Only the researchers involved in the study had access to the subjects’ personal medical records. Data will be processed anonymously, omitting information that can identify subjects individually, and the results will be published without disclosing subjects’ personal information.

The Inclusion criteria was as follows: age < 15 years; American Society of Anesthesiologists (ASA) class II; Children with ductus arteriosus greater than 2 mm in diameter or not closed on their own after three years old; underwent robot-assisted thoracoscopic closure of PDA during day surgery; and the time from admission to discharge was within 24 h. Exclusion criteria: transferred to general ward for treatment for over 24 h due to complications such as pulmonary infection or pneumothorax, which quitted the PDA day-case surgery process.

The following principles have been followed before the surgeries, to maximize the benefits and minimize complications of day-case surgery: (1) A low risk of major post-operative complications that necessitate immediate intervention, such as hemorrhage or cardiovascular instability; (2) Urgent procedures, such as abscess drainage and certain trauma surgeries, may be appropriate for day-case cases; (3) Prolonged post-operative specialist care or observation should not be required; (4) Minimally invasive techniques should be used for opening the abdominal and thoracic cavities; (5) Post-operative pain can be managed with oral analgesics, with or without regional anesthesia; (6) Patients should be able to resume normal functions, like oral nutrition and safe mobilization, rapidly; (7) Anesthesia-related side effects that delay discharge, such as post-operative nausea, vomiting, drowsiness, or urinary retention, must be minimal [5].

Indications of anesthesia for pediatric day surgery are followed according to American Society of Anesthesiologists (ASA): For patients of ASA Grade I and II, small changes in physiological function during and under anesthesia are expected, and the incidence of postoperative complications is expected to be low. Anesthesia is not recommended for pediatric day surgery in the ASA grade III and IV; Postgestational age < 60 weeks (preterm infants are older than normal newborns); the expected operation time > 2 h; Severe postoperative pain, bleeding, or delayed dyskinesia; Serious intraoperative complications due to underlying or existing medical conditions; Children with recent acute upper respiratory tract infection, asthma attack and ongoing status, uncontrolled epilepsy; Airway problems such as small jaw; Estimated long recovery time of postoperative respiratory function pathological obesity or sleep apnea syndrome; Untreated myocardial ischemia or arrhythmia; Children with untreated right-to-left shunt heart disease; Children who stopped taking monoamine oxidase inhibitors for less than 10 days were not accompanied by an adult for 24 h after discharge. Abnormal preoperative examination, such as hemoglobin (Hb) < 70 g/L, platelet (PLT) < 100 × 109/L, coagulation time (PT) extended by 8 s, activated partial thromboplastin time (APTT) extended by more than 8 s or fibrinogen (FIB) < 2 g/L, blood potassium (K+) < 3mmol/L, Blood sodium (Na+) < 130mmol/L.

### Preoperative preparation

Within 1 week before the surgery, complete blood routine, coagulation function, liver and kidney function, electrolyte, electrocardiogram, chest X-ray, and echocardiogram examinations were performed, and preoperative evaluation and surgical appointment were conducted in the anesthesia outpatient department. On the day of surgery, the anesthesiologist evaluated the patient’s overall condition again and signed the informed consent for anesthesia. The preoperative fasting time was 8 h for fried and meat foods, 6 h for formula milk and starchy foods, 4 h for breast milk, and 2 h for clear drinks. Two hours before the surgery, 5 ml/kg of preoperative energy supplement (carbohydrate drink) was given, and 0.5 mg/kg of midazolam oral solution was given 30 min before the surgery.

### Anesthesia method

Upon entering the operating room, routine monitoring was performed, including heart rate (HR), noninvasive blood pressure (NBP), electrocardiogram (ECG), and oxygen saturation of pulse (SpO_2_). Anesthesia induction was performed with etomidate at a dose of 0.3 mg/kg, rocuronium at a dose of 0.6 mg/kg, fentanyl at a dose of 5 µg/kg, and methylprednisolone succinate at a dose of 2 mg/kg. After jaw relaxation, tracheal intubation was performed, and the position of the endotracheal tube was confirmed by auscultation of lung sounds. The pressure-controlled ventilation volume-guaranteed (PCV-VG) mode was set, with an FiO_2_ of 0.4, a tidal volume (Vt) of 6–8 ml/kg, a respiratory rate of 15–25 breaths/min, a positive end-expiratory pressure (PEEP) of 5 cmH_2_O, and a quantitative end-tidal CO_2_ (P_ET_CO_2_) < 60 mmHg in the case of pneumothorax. After anesthesia induction, a radial artery catheter was placed for monitoring arterial blood pressure, and a femoral vein catheter was placed for rapid blood and fluid infusion in emergency situations. The oral temperature was monitored during the surgery. If the body temperature was lower than 36 °C, a warm air blower and infusion warmer were used to maintain a temperature above 36 °C. During the surgery, remifentanil was infused at a rate of 0.5 µg·kg-1·min-1, and 2–3% sevoflurane was inhaled to maintain anesthesia.

Before PDA ligation, 1-3 µg/kg of remifentanil was administered intravenously, and the concentration of sevoflurane was adjusted to 3-5% to reduce the mean arterial pressure (MAP) to around 55 mmHg. After PDA ligation, a transthoracic echocardiogram was performed to confirm the successful ligation of PDA without residual shunt. Before skin closure, lung inflation was performed adequately to remove pneumothorax. The incision was infiltrated with 0.3% ropivacaine, and ondansetron (0.1 mg/kg) was administered intravenously to prevent nausea and vomiting. Before extubation, sugammadex (2 mg/kg) was administered to antagonize the muscle relaxation effect of rocuronium. The endotracheal tube was removed when the tidal volume was > 6 ml/kg, the respiratory rate was > 15 breaths/min, the P_ET_CO_2_ was normal, and the deoxygenated SpO_2_ was ≥ 95%. After stable vital signs were confirmed, S-ketamine (0.2 mg/kg) was administered intravenously for postoperative analgesia. The patient was then transferred to the post-anesthesia care unit (PACU) for observation. When the Steward recovery score was ≥ 4, the patient was sent back to the day ward [9]. According to Expert consensus on clinical application of perioperative lung-protective ventilation strategies in 2020 [10], the rising rate of PaCO2 should be controlled to < 10mmHg/h, the PaCO2 should be lower than 65mmHg, the pH value should be higher than 7.20. Optimal oxygen method [10]: Start by setting a PEEP of 3 to 5cmH2O, and increase the PEEP by 2 to 3cmH2O each time according to the oxygenation situation. When FiO2 ≤ 0.6, the PEEP that meets PaO2 ≥ 60mmHg or PaO2/FiO2 ≥ 300mmHg is the best PEEP. Whether the first ideal PEEP is 5cmH20, should be determined individually in the clinical practice. In the management of clinical anesthesia, various factors should be considered comprehensively to exert the best protective effect.

### Surgical procedure

The patient was placed in a right lateral position with the right arm elevated, and the position was secured. A trocar was inserted at the fifth intercostal space of the left axillary midline, and artificial pneumothorax was established by inflation to a pressure of 4 mmHg. Under direct vision, operating trocars were placed at the fourth intercostal space of the left anterior axillary line and the sixth intercostal space of the scapular line for dissection. If the surgical field of view was poor, an auxiliary trocar was placed at the seventh intercostal space of the left anterior axillary line for traction and exposure. Using the fourth-generation Da Vinci robotic system (Intuitive Surgical, Inc. Sunnyvale, CA USA), the patent ductus arteriosus was separated to avoid damaging the vagus nerve branches, and after controlled hypotension, the ductus arteriosus was ligated with double 7 − 0 silk sutures. It allows surgeons to perform operations on children in a smaller operating space and with higher operating accuracy requirements [11].

### Postoperative discharge and follow-up

The patient started early ambulation and gradually progressed from clear fluids to solid foods. Before discharge, blood routine, chest X-ray, and temperature were re-examined, with a temperature of less than 38 °C to rule out pulmonary infection and pneumothorax. The Pediatric Post Anesthetic Discharge Scoring System (Ped-PADSS) [12] was used to assess the patient’s post-anesthesia discharge readiness, with a score of 9 or higher considered suitable for discharge. Antibiotics were prescribed after discharge to prevent respiratory infections, and the chest incision was dressed and changed the medication once every 2 days until the scabbed. Blood routine was rechecked three days after discharge, and outpatient follow-up and echocardiography were performed one month after discharge. According to Ped-PADSS (Table 1), the children can be discharged when the total scores of all items are ≥ 9.

**Table 1:** The pediatric post anaesthetic discharge scoring system (Ped-PADSS)

Ped-PADSS		
Items	Indicators	Scores
Vital sign	Bp and pulse fluctuated 20% based on pre-operation	2
	Bp and pulse fluctuated 20-40% based on pre-operation	1
	Bp and pulse fluctuated over 40% based on pre-operation	0
Activities	The gait was stable without dizziness or reached the preoperative level	2
	Need assistance	1
	immobility	0
Nausea and vomiting	Mild/oral medication	2
	Moderate/injectable drug treatment	1
	Moderate/persistent despite medication	0
Pain	Oral medication is administered to the patient at an acceptable level of analgesia	
	Yes	2
	No	1
Surgical bleeding	Mild/no dressing change required	2
	Moderate/Need 2 dressing changes	1
	Severe/need to change dressing ≥ 3 times	0

Unplanned readmission of patients is generally related to postoperative hypoxemia, nausea, vomiting, pain, bleeding, pulmonary infection, pneumothorax, and other surgical complications. Post-anesthesia education was provided, and hospital contact information was provided. Within 24 h, a nurse with strong communication skills routinely followed up with the patient via phone to inquire about any anesthesia- or surgery-related complications and provide treatment advice.

The patient’s basic information, such as gender, age, weight, PDA diameter, and hospitalization costs, was recorded as part of the observation indicators. Intraoperative conditions were also observed, including surgery time, anesthesia time, extubation time, intraoperative blood loss, MAP before pneumothorax, HR before pneumothorax, PaCO_2_ before pneumothorax, MAP during controlled hypotension, HR during controlled hypotension, and PaCO_2_ during controlled hypotension. Postoperative conditions were monitored, such as PACU stay time, agitation during the recovery period, pain, and the incidence of nausea and vomiting. After discharge, the recovery status was assessed, including the unplanned revisit rate within 30 days of discharge, unplanned readmission rate, surgical wound infection rate, and residual shunt rate on echocardiography 1 month after the procedure.

The assessment tool for the children was Face Legs Activity Cry and Consolability (FLACC) [13]: It is used for pain assessment in children aged 2 months to 7 years, but it is also used in pain assessment in children aged 0–18 years. The pain Behavior Scale (FLACC) was used to score the pain, which included 5 behavioral changes: facial expression, leg movement, body position, crying and comfortability. The score for each item was 0 ~ 2 points, and the total score was 0 ~ 10 points. The higher the total score was, the more severe the pain was. Score 0: relaxed, comfortable; 1–3: mild discomfort; 4–6: moderate pain; 7–10: severe pain, discomfort, or both. The higher the score, the more severe the pain.

### Statistical analysis

Statistical analysis was performed by SPSS 21.0 software. The t test compared the means of two independent groups in the indicators such as basic information, intraoperative conditions and recovery status after discharge, in order to determine whether there is statistical evidence that associated the means is significantly different. *P* < 0.05 was considered to be statistically significant.

## Results

### General clinical information

In the study, 70 out of 72 children successfully completed the surgery and were discharged within 24 h. Two cases (1 case of postoperative respiratory tract infection and 1 case of postoperative pneumothorax) were transferred to a general ward for further treatment. Of the 70 children, 27 were male and 43 were female, with ages ranging from 11 to 82 months, weights ranging from 8.0 to 25.0 kg, PDA diameters ranging from 1.4 to 4.2 mm, and hospitalization costs ranging from 49,492 to 53,600 yuan. The surgery time ranged from 43 to 76 min, anesthesia time ranged from 65 to 118 min, extubation time ranged from 5 to 16 min, and PACU stay time ranged from 15 to 32 min. The intraoperative blood loss was between 1 and 4 ml (Table 2).

**Table 2:** General clinical information, median (range)

Indexes	Clinical data
Male/Female (cases)	27/43
Age(month)	28(11 ~ 82)
Body weight(kg)	11.9(8.0 ~ 25.0)
PDA diameter(mm)	2.5(1.4 ~ 4.2)
Operating time(min)	50(43 ~ 76)
Anesthesia time(min)	83(65 ~ 118)
PACU stay(min)	22(15 ~ 32)
Bleeding volume(ml)	1.5(1 ~ 4)
Extubation time(min)	8(5 ~ 16)
Hospitalization cost(yuan)	51,615(49,492 ~ 53,600)

### Comparison of MAP, HR, and PaCO2 before pneumothorax and during controlled hypotension

The MAP before pneumothorax was 69.58 ± 12.52 mmHg, while the MAP during controlled hypotension was 54.96 ± 15.23 mmHg (*P* < 0.001, Table 3). The HR before pneumothorax was 109.00 ± 21.10 beats/min, while the HR during controlled hypotension was 102.12 ± 22.10 beats/min. The PaCO2 before pneumothorax was 38.69 ± 3.38 mmHg, while the PaCO_2_ during controlled hypotension ranged was 51.42 ± 4.05 mmHg (*P* < 0.001, Table 3).

**Table 3:** Comparison of MAP, HR, and PaCO2 between before pneumothorax and during controlled hypotension

Indexes	Before pneumothorax	During controlled hypotension.	*t*	*P*
MAP(mmHg)	69.58 ± 12.52	54.96 ± 15.23	5.715	< 0.001
HR(beat/min)	109.00 ± 21.10	102.12 ± 22.10	1.711	0.089
PaCO2(mmHg)	38.69 ± 3.38	51.42 ± 4.05	25.034	< 0.001

### Postoperative adverse reactions and complications

No incidence of reintubation after planned extubation was found. Three cases experienced agitation during the recovery period, and four cases experienced mild pain. No cases of moderate or severe pain or nausea and vomiting were reported. The unplanned revisit rate within 30 days of discharge, unplanned readmission rate, and surgical wound infection rate were all 0. The residual shunt rate on echocardiography 1 month after the procedure was also 0 (Table 4).

**Table 4:** Postoperative adverse reactions and complications [Cases (%)]

	Cases number (%)
Restlessness during the waking period	3(4.3%)
Mild pain	4(5.7%)
Moderate to severe pain	0(0%)
Nausea and vomiting	0(0%)
Unplanned readmission within 30 d of discharge	0(0%)
Unplanned readmission within 30 d of discharge	0(0%)
Surgical wound infection	0(0%)
Residual PDA shunt after 1 month	0(0%)

## Discussion

Day-case surgery refers to a surgical model in which patients complete hospitalization, surgery, postoperative observation, recovery, and discharge within one working day (24 h). Although day-case surgery has many advantages such as shortening hospitalization days, avoiding hospital-acquired infections, shortening hospital waiting times, improving bed turnover rate, at the same time, PDA ligation is a tertiary surgery with inherent complexity. Day-case surgery for paediatric PDA ligation necessitates more careful anesthesia management [14].

### Da Vinci robot

Compared with adults, pediatric chest space is small, and traditional open-chest PDA ligation is limited and more traumatic [15]. The progress of laparoscopic surgery also has some drawbacks, such as two-dimensional views, restricted ergonomics for surgeons, and limited range of motion of surgical instruments [16]. Moreover, thoracoscopic PDA ligation still requires single-lung ventilation to obtain better visibility, which limits the operation and results in a high incidence of postoperative pulmonary atelectasis [17].The fourth-generation Da Vinci robotic system used in this day-case surgery study has a clear and accurate magnified three-dimensional view, an ergonomic operating console, and real-time accurate conversion of the operator’s finger and wrist movements into mechanical actions [11]. The range of instrument bending and rotation exceeds the limit of human hand movements, and the strong tremor filtering function allows stable and precise operation with both hands. In all cases, single-lung ventilation was not required during surgery, effectively reducing the incidence of postoperative pulmonary atelectasis caused by single-lung ventilation [11].

### Preoperative sedation

Pediatric patients are prone to fear and anxiety before surgery, which can lead to prolonged anesthesia induction time, longer stay in the PACU, increased consumption of analgesics, more complications, and prolonged wound healing time [18]. Oral administration of 0.5 mg/kg midazolam has good anxiolytic and amnestic effects, with a short duration of action, high acceptance by pediatric patients, and helps with the separation of the child from their parents [19].

### Fasting and preoperative consumption of surgical energy within 2 h before surgery

Traditional prolonged fasting can have various adverse effects on pediatric patients, such as thirst, hunger, dehydration, and hypoglycemia. Currently, experts agree on the following recommendations: fasting solid food 6 h before surgery, fasting breast milk 4 h before surgery, refraining from clear fluids 2 h before surgery, and consuming liquid rich in carbohydrates (surgical energy) within 2 h before surgery is beneficial for the patient’s postoperative recovery [18]. Studies have shown that drinking a carbohydrate drink 2 h before surgery does not cause delayed gastric emptying and reduces postoperative insulin resistance, which can improve patient satisfaction and clinical outcomes [19–23]. Preoperative consumption of Outfast 5 ml/kg (Yichang Humanwell Pharmaceutical Co., ltd. China) within 2 h before surgery in this study, can relieve preoperative hunger, thirst, and anxiety, without increasing the risk of reflux or aspiration during surgery, thus, accelerating patient recovery, and shortening hospital stay.

### Controlled hypotension and fluid replacement

Controlled hypotension is performed before PDA ligation to reduce the mean arterial pressure (MAP) to around 55mmHg, which reduces the difficulty of the ligation procedure and prevents the risk of accidentally cutting the ductus arteriosus due to high pressure. Nitroprusside is a potent vasodilator and commonly used in pediatric perioperative management for controlled hypotension. A study has shown that a combination of remifentanil and sevoflurane can be used for controlled hypotension in children, reducing middle ear blood flow (MEBF) and providing good surgical conditions for middle ear surgery without the need for additional hypotensive drugs [24]. In this study, remifentanil was administered at a dose of 1-3µg/kg intravenously, and a continuous infusion of 0.5 µg·kg-1·min-1 was used, while sevoflurane concentration was adjusted to 3%~5%. The infusion rate of remifentanil and the concentration of sevoflurane were both commonly used in pediatric anesthesia maintenance, showing satisfactory efficacy and safety without the use of additional potent hypotensive drugs. As daytime surgery is relatively short, less traumatic, and the general condition of the patient is good, preoperative oral intake of Outfast for 2 h is used for fluid and energy supplementation, while a restrictive fluid replacement strategy is used during surgery, and early postoperative feeding is implemented to restore the patient’s fluid balance [25].

### Mechanical ventilation mode

The mechanical ventilation mode chosen by our team is PCV-VG mode, which combines the advantages of VCV mode and PCV mode. It achieves the target tidal volume with the lowest airway peak pressure while ensuring adequate ventilation. The airway pressure increases to its maximum value at the start of inspiration and remains there during the whole inspiratory phase due to the PCV-VG mode’s decreasing gas flow [26]. As a result, tiny airways and alveolar tissue can open as quickly as feasible, allowing even low compliance tissue to get some ventilation. The danger of harm brought on by high airway pressure is decreased by the continuous plateau pressure, which is more favorable to oxygen diffusion. It can also improve pulmonary shunting and compliance to some extent, and is beneficial for alveolar ventilation and oxygenation [27]. At the same time, to avoid lung injury caused by high volume and high airway pressure, provide a clear view for the surgeon and reduce postoperative complications such as pneumothorax.

### Lung protective ventilation strategies

Lung Protective Ventilation Strategies (LPVS) primarily includes low tidal volume, moderate positive end-expiratory pressure (PEEP), permissive hypercapnia (PHY), lung recruitment, and low inspired oxygen concentration.

Reducing lung ventilation drive by using low tidal volume is the basis of LPVS strategy. Traditional mechanical ventilation tidal volume is usually 10–15 ml/kg, far higher than the normal tidal volume of the body, which can lead to overinflation of lung tissue, excessive expansion of alveoli and high pressure, resulting in pressure-volume and inflammatory lung injury [28]. Therefore, a tidal volume of 6–8 ml/kg (ideal body weight) is recommended. Low tidal volume ventilation may lead to CO2 accumulation, which in turn causes hypercapnia [29]. Currently, it is believed that a certain range of hypercapnia, namely permissive hypercapnia, can reduce ischemia-reperfusion injury, reduce oxidative stress response, increase cardiac output, improve arterial oxygen pressure, reduce pulmonary shunting, and play a protective role in the lung. However, PHY should be maintained within a certain limit, otherwise it may cause internal environment disorder [28]. Research suggests that the rate of PaCO_2_ rise should be controlled to < 10 mmHg/h, PaCO_2_ should be < 65 mmHg, and pH should be > 7.20 [30]. Therefore, in clinical anesthesia management, various factors should be considered comprehensively to achieve the best protective effect.

If PEEP is too low, it is not enough to maintain the open state of alveoli, leading to alveolar collapse and atelectasis. If PEEP is too high, it can cause an increase in airway pressure, over-distention of normally ventilated lung tissue, and exacerbate lung injury. A meta-analysis of trials using high (8–15 cm H_2_O) versus low (2–5 cm H_2_O) PEEP showed no significant difference in postoperative complications, length of hospital stay, or mortality rates, and the group with PEEP ≥ 5 cm H2O had fewer postoperative pulmonary complications compared to the group with PEEP = 0 cm H2O [31]. In this case, a PEEP of 5 cm H_2_O was applied to prevent alveolar collapse.

During mechanical ventilation, using a lower fraction of inspired oxygen (FiO_2_) of 0.4 can help reduce absorption atelectasis and maintain continuous opening of the alveoli [30]. If persistent hypoxemia occurs, increasing FiO2 or adjusting PEEP can help improve oxygenation. In this case, a “lung-protective ventilation strategy” was employed, including a low tidal volume (Vt 7 ml/kg), moderate positive end-expiratory pressure (PEEP in cm H_2_O), permissive hypercapnia (PaCO_2_ 45–60 mmHg), and low FiO_2_ (0.4).

### Waking period

The prolonged duration of action and residual paralysis of non-depolarizing muscle relaxants are more common in children. Residual paralysis can lead to respiratory system complications such as early airway obstruction, hypoxemia, and bronchospasm after extubation [32]. Studies have shown that compared to neostigmine, using sugammadex to antagonize rocuronium-induced muscle relaxation in pediatric tonsillectomy not only shortens the time of extubation and PACU stay but also effectively reduces the incidence of early postoperative respiratory complications [33]. In this study, the use of sugammadex (2 mg/kg) to antagonize rocuronium-induced muscle relaxation is helpful for early extubation and smooth recovery of children undergoing day-case surgery. Children with a Steward score ≥ 4 can leave the PACU.

### Multimodal analgesia

To provide additive (or even synergistic) pain relief while resulting in an opioid-sparing effect and limiting side effects, combinations of various medicines having different mechanisms of action and/or operating at different sites along the pain pathway are used [34, 35]. As the effectiveness of multimodal analgesia regimens continues to improve, opioid drugs are gradually used only for rescue analgesia, rather than as the main postoperative treatment. The side effects of opioid drugs include nausea, vomiting, intestinal obstruction, urinary retention, delirium, drowsiness, and respiratory depression, all of which are detrimental to postoperative recovery and can delay discharge [36]. S-ketamine has strong sedative and analgesic effects. It is the right-handed isomer of S-ketamine and is twice as potent as the racemic mixture, enhancing analgesic effects while producing fewer side effects than the racemic mixture [37]. In this group, 0.3% ropivacaine local infiltration combined with S-ketamine 0.2 mg/kg intravenous injection was used for postoperative analgesia, reducing the complications caused by postoperative opioid drugs and the incidence of restlessness during the recovery period in children.

### Day-case PDA efficiency in children

No moderate or severe pain, nausea, or vomiting occurred among the children in this study. After discharge, the children had unplanned revisit rate, unplanned readmission rate. These results showed the day-case surgery had the advantages of short operation time and few complications, as reported [4, 5]. Furthermore, the robotic assistance helped reduce the pain and revisit rate of the children, improved the recovery of the children [8]. Since there were no reports of day-case PDA surgeries among children, the results of this study may provide some experiences for day case pediatric PDA. However, there was still 1 case of postoperative respiratory tract infection and 1 case of postoperative pneumothorax, which suggested the risk of surgeries still exist. Close postoperative care is still needed.

A comparison report found that the 105 patients with device closure had an higher average cost than the 96 patients with surgical closure [38]. Ying et al. [8] also reported the average hospitalization costs with robotic assistance were much more expensive than those patients who received transcatheter procedure. In this study, hospitalization costs ranging from 49,492 to 53,600 yuan, which might also be expensive. The guardians chose robotic assisted day-case PDA surgery for their children, so that the children may have little pain and few complications regardless of the high cost.

### Limitation of this study

The study was lacking in data concerning long-term outcomes such as the rate of PDA recurrence or the need for additional interventions. Furthermore, this study was limited for its small sample size, and forthcoming studies must feature a more substantial sample size, a more elaborate methodology section, and a more extensive range of results information. At last, the comparison with open thoracotomy nor VAT without robot was not studied in this study, which may need further explorations.

## Conclusion

The robot-assisted thoracoscopic surgical system lead to limited trauma, bleeding, and postoperative pain, with still a possibility of respiratory infection and pneumothorax in the children who received day-case PDA surgeries. With reasonable preoperative preparations, precise anesthesia management, and comprehensive postoperative follow-up, day-case PDA surgeries under robot-assisted thoracoscopic closure can be implemented, reduce the complications, and to improve the comfort of the child.

## Data Availability

All data analysed during this study are included in this published article.

## List of abbreviations

PDA: patent ductus arteriosus
PaCO_2_: partial pressure of carbon dioxide
